# Supplementary material for: 137Cesium (137Cs) assessment in wild boars from northwestern Italy
Source: PLoS One. 2024 May 9;19(5):e0303093. doi: 10.1371/journal.pone.0303093 (PMC11081301; doi:10.1371/journal.pone.0303093)
Supplement: Attachment — Submitted filename: Answers To Reviewers 2023 09 07 variato.docx [file pone.0303093.s003.docx]

Dear Editor,

below you will find the comments and answers to the previous version of the work.

We would thank you for the time dedicated to our manuscript titled “Radionuclides assessment in wild boars from Northwest Italy”.

We would thank reviewer 1 and 2 for their comments that have helped us to improve the quality of our work.

For us it is a great opportunity to improve the content and the style of our work according to the comments provided by the reviewers. We have answered to every concern point by point.

Please consider it as a new submission considering the new statistical analysis were done.

**Comments by Reviewer 1**

Reviewer #1:

Comment:

Although the study appears to be sound and the topic is interesting, the language is unclear, making it difficult to follow. I advise the authors to review the writing carefully to get a good flow. Free applications like “Grammarly” could be used to help with grammar issues and to improve clarity. i.e text from lines 41 to 45 could be rewritten as: “Radionuclides contamination is becoming a relevant/significant/serious issue for human health. It comprises an anthropogenic source of contamination derived from nuclear experiments and accidents at nuclear power plants, with a potentially dramatic impact, like in the cases of Chornobyl and Fukushima.

Answer:

The text has been revised by a mother tongue reviewer and the certificate is included in the submission. The suggestion has been included in the text (Page 2 from line 24 to line 28)

Comment:

Furthermore, the information in the different sections should be thoroughly reviewed since they have been mixed several times. Eg: In line 169 of “Results and Discussion”, the first paragraph describes materials and methods and should be included in the corresponding section. These are not results. The text from line 177 to line 183 could be included in the introduction, to avoid repeating concepts that have already been explained in the previous section. This also happens in line 184; the authors are explaining the results in the Materials and methods section. Again, in line 237, materials and methods are stated.

Answer:

The text has been rewritten following the suggestions and the paragraph have been moved in different sections o deleted if repeated as suggested (*e*.*g*.: Page 7 from line 156 to line 160, Page 4 from line 75 to line 81, from Page 7 line 162 to Page 8 line 166)

**Comment**:

Regarding statistical analysis, I could not completely understand what the authors did. Bland Altman plots should be included in the figures since they were used to achieve the second objective of the present study. The materials and methods should be explained in detail, since the text is difficult to follow.

**Answer**:

In the revised version of the manuscript, statistical analysis was completely redone, following the Reviewers’ comments, which pointed out lack of clarity in the study objectives and in the adopted methods. In fact, in the first version of the manuscript, we used the Bland Altman plots to focus on concordance agreement among measurement on different organs. Whereas we now look at the data from another, more straightforward perspective, the main focus being on comparison among concentrations of Cs137 in different wild boar organs. To achieve such an objective, we used a statistical regression model, considering distribution of the original data, as well as the correlation structure of observations, arising from repeated measurements from the same wild boars. We also tested whether greatest Cs137 levels in certain organs would be affected by wild boar age, by adding interaction terms. We believe that, in this way, results and subsequent conclusions are much clearer and useful for further research and practical applications. We, therefore, thank the reviewers for helping in this process, to improve the manuscript (From Page 8 line 180 to Page 9 line 210).

**Comment**:

Explain the criteria used to select the organs in which Cs values were determined.

**Answer**:

The organs were chosen from existing literature and considering the main body systems (page 8 from line 165 to line 170)

**Comment**:

The reasons for Log transformation of the data should be stated.

**Answer**:

Testing and departure from normality are reported in the revised version of the manuscript (Page 9 from line 193 to line 201).

**Comment**:

The statistical software used to analyse the data should be reported.

**Answer**:

Software and procedures used in statistical analysis are reported in the revised version of the manuscript (in the text from Page 8 line 180 to Page 9 line 210).

**Comment**:

I cannot ascertain if the statistical analysis was correctly performed since I am not sure I correctly understood the procedure.

**Answer**:

The main statistical analysis is now based upon a well-established and well-known mixed model technique, and detailed explanations and justifications of the analysis are now provided (Page 8 line 180 to Page 9 line 210).

**Comment**:

Normally, to homogenize the results (contamination levels in each organ), the mean and not the median is employed.

**Answer**:

Testing and departure from normality are reported in the revised version of the manuscript, therefore median and quartiles are chosen as most appropriate in descriptive statistics of non-normal data (Page 8 from line 180 to line 186).

**Comments by Reviewer 2**

**Comment**:

Very interesting and important issue affecting animal and public health. There is a lack of knowledge and surveillance over pollutants (especially from anthropogenic origin) present in wildlife, particularly those species consumed by humans that are entering into the food chain. Heavy metals are more known by society but radionuclides are generally unknown and neglected, therefore any effort focused on increasing the knowledge of the current environmental situation and definition of bioindicators are very important.
While the research topic and content are proper for publishing in this journal, due to the limited study area I considered the publication of this manuscript more appropriate as a short communication, after an extensive review throughout the manuscript has been done by the authors. In the current state, grammar can be improved and some sentences and paragraphs in the manuscript should be rewritten as they are quite difficult to understand.

Answer: the text has been revised by a mother tongue reviewer and the certificate is included in the submission.

Comment: L82-83 why are radionuclides gaining attention? As it is, this sentence does not provide any information.

**Answer**:

The sentence has been rewritten and, in the introduction, more explanations are given (Page 4 line 67 and *e*.*g*. page 5 from line 98 to line 102).

Comment: L106-117 It looks like the authors are avoiding the use of the term bioindicator, when it is the correct term to describe those organisms providing us an idea of the health of an ecosystem.
Answer: the paragraph has been deeply revised and the terms “bioindicator” and “hyperaccumulator”, as indicated in literature (Piñero-García F, Thomas R, Mantero J, Forssell-Aronsson E, Isaksson M. (2022). Concentration of radionuclides in Swedish market basket and its radiological implications. *Food* *Control*, 133, 108658. <https://doi.org/101016/j.foodcont.2021.108658>) (Page 5 from line 103 to line 111).

**Comment**:

The Material and Methods section lacks a lot of information.

**Answer**:

The section was rewritten and completed (Form Page 7 line 152 to Page 8 line 179)

**Comment**:

What is the age-sex distribution of the wild boars used in the study?

**Answer**:

The requested information is provided in the revised version of the manuscript (Page 7 from line 156 to line 161 and Page 8 line 164).

**Comment**:

Which statistical test was used to evaluate differences between sex, valleys and years?

**Answer**:

As clarified in the revised version of the manuscript, non-parametric tests, such as Wilcoxon two-sample test, and Kendall’s tau were used in preliminary analysis. The association between wild boar organs and the natural log of C137 concentration, and organ-by-age interaction were tested by a mixed regression model, as explained in the answer to the first comment of Reviewer 1. Wild boar sex was also included in the regression model, but it was not associated with the natural log of C137 (From page 8 line 180 to Page 9 line 210).

**Comment**:

The authors state that not statistical differences were found but results are difficult to find in the Results and Discussion section with not statistical information provided (e.g., p value).

**Answer**:

Statistical analysis was completely redone and a different approach was adopted, in comparison with the first version of the manuscript. All details and justification, including clear results are, hopefully, provided in the revised version of the manuscript (Results and Discussion section from Page 9 line 211 to Page 14 line 326: *e*.*g.* Page 10 from line 222 to line 225, Page 11 from line 251 to line 255) and Table 2.

**Comment**:

I understand that the authors decided to use the median instead of the mean (L248) for statistical analysis and comparations, right? Is there any rational behind this decision? Please explain.

**Answer**:

Testing and departure from normality are reported in the revised version of the manuscript, therefore median and quartiles are chosen as most appropriate in descriptive statistics of non-normal data, since these descriptive statistics are not affected by outliers and homogeneous observations (Page 8 from line 180 to line 186).

**Comment**:

L169-174 belongs to Introduction and, as a matter of fact, it is a better version of the objectives of the study than the provided in L118-123 and L130-136.

**Answer**:

The part has been moved to the introduction section and the other paragraph has been deleted (Page 7 from line 143 to line 150).

**Comment**:

L174-176 belongs to Material and Methods section.

**Answer**:

The paragraph has been moved to the Materials and methods section (Page 7 from line 156 to line 157)

**Comment**:

L177-182 belongs to introduction section.

**Answer**:

The paragraph has been moved to the Introduction Section (Page 4 from line 75 to line 81 and Page 5 from line 103 to line 111)

**Comment**:

The maximum level permitted (L197) as well as the reported contamination in other European countries should go in the Introduction section as a framework of the situation and one of the reasons to carry out the present study.

**Answer**:

The paragraph has been moved to the introduction section (Page 5 from line 86 to line 93)

**Comment**:

Only in the Conclusions section the authors state the importance of radionuclides as an important source of contamination due to nuclear plants. Until this moment nuclear plants were only mentioned as a source of contamination in the event of an accident (e.g., Chernobyl and Fukushima). If the normal operation of nuclear power plants produces radionuclides, then the importance of this type of studies increases and should be stated in the Introduction.

**Answer**:

At the present state contamination sources have been identified as specified in the text: natural presence of radioactive elements, nuclear weapons test or use and nuclear plants accidents as stated in literature (Aliyu AS, Evangeliou N, Mousseau TA, Wu J, Ramli AT. (2015). An overview of current knowledge concerning the health and environmental consequences of the Fukushima Daiichi Nuclear Power Plant (FDNPP) accident. *Environ* *Internat* 85, 213-228; Vetikko V, Kostantiainen E. (2013). Assessment of doses to game animals in Finland. *J* *Enviro* *Radioact*, *125*, 69-73).
