# Supplementary material for: 137Cesium (137Cs) assessment in wild boars from northwestern Italy
Source: PLoS One. 2024 May 9;19(5):e0303093. doi: 10.1371/journal.pone.0303093 (PMC11081301; doi:10.1371/journal.pone.0303093)
Supplement: Attachment — Submitted filename: Manuscript PONE-D-23-24864R Response to reviewers 2024 04 11.doc [file pone.0303093.s005.doc]

Dear Editor and Reviewers,

For the second time we want to thank you very much for the time dedicated to our revised manuscript. We worked on the suggestions in order to improve the comprehension of the text and to be easier to follow the text. We agree that in the previous version of the manuscript, there were other concerns to resolve, and we truly thank you and the editorial office for the opportunity to answer to all issues arising from the review process by you and by the referees.

The answers are written in red, whereas the changes in the manuscript are highlighted in yellow Enclosed here, you find all the changes and the additions we made to answer the questions raised in the revision process.

PONE-D-23-24864R1

137Cesium (137Cs) assessment in wild boars from northwestern Italy

PLOS ONE

Dear Dr. pattono,

Thank you for submitting your manuscript to PLOS ONE. After careful consideration, we feel that it has merit but does not fully meet PLOS ONE’s publication criteria as it currently stands. Therefore, we invite you to submit a revised version of the manuscript that addresses the points raised during the review process.

Please submit your revised manuscript by Apr 20 2024 11:59PM. If you will need more time than this to complete your revisions, please reply to this message or contact the journal office at [plosone@plos.org](mailto:plosone@plos.org). When you're ready to submit your revision, log on to <https://www.editorialmanager.com/pone/> and select the 'Submissions Needing Revision' folder to locate your manuscript file.

Please include the following items when submitting your revised manuscript:

- A rebuttal letter that responds to each point raised by the academic editor and reviewer(s). You should upload this letter as a separate file labeled 'Response to Reviewers'.
- A marked-up copy of your manuscript that highlights changes made to the original version. You should upload this as a separate file labeled 'Revised Manuscript with Track Changes'.
- An unmarked version of your revised paper without tracked changes. You should upload this as a separate file labeled 'Manuscript'.

If you would like to make changes to your financial disclosure, please include your updated statement in your cover letter. Guidelines for resubmitting your figure files are available below the reviewer comments at the end of this letter.

If applicable, we recommend that you deposit your laboratory protocols in [protocols.io](http://protocols.io/) to enhance the reproducibility of your results. Protocols.io assigns your protocol its own identifier (DOI) so that it can be cited independently in the future. For instructions see: [https://journals.plos.org/plosone/s/submission-guidelines#loc-laboratory-protocols](https://journals.plos.org/plosone/s/submission-guidelines#loc-laboratory-protocols). Additionally, PLOS ONE offers an option for publishing peer-reviewed Lab Protocol articles, which describe protocols hosted on [protocols.io](http://protocols.io/). Read more information on sharing protocols at <https://plos.org/protocols?utm_medium=editorial-email&utm_source=authorletters&utm_campaign=protocols>.

We look forward to receiving your revised manuscript.

Kind regards,

Mohamad Syazwan Mohd Sanusi
Academic Editor
PLOS ONE

Journal Requirements:

Please review your reference list to ensure that it is complete and correct. If you have cited papers that have been retracted, please include the rationale for doing so in the manuscript text, or remove these references and replace them with relevant current references. Any changes to the reference list should be mentioned in the rebuttal letter that accompanies your revised manuscript. If you need to cite a retracted article, indicate the article’s retracted status in the References list and also include a citation and full reference for the retraction notice.

Additional Editor Comments:

Decision: Minor Revision

General Comments: The title of the work, "137Cesium (137Cs) assessment in wild boars from northwestern Italy," is worthy of study. The proposed method, with large samplings and targeted organ samples of the wild boars (48 for liver and 16 samples for each of the 5 types of samples), is technically sound. The method is sufficient, and the qualitative analysis approach investigating the correlation between Cs levels and age, and different types of tissue organs, seems appropriate in the field of radioecology. However, the manuscript's results and discussion are difficult to follow, especially when the author mixes up the results and discussion.

(1) Therefore, it is suggested that the author separate the results and discussion to make it easier and more comprehensible for readers to follow.

Answer

The results and the discussion were separated in two different sections in order to improve comprehension and make the paper easier to follow (from Page 11 Line 235 to Page 19 Line 423).

(2) The presentation of the results also seems insufficient. As most of the results are not directly presented in a table and mostly discussed in the text, I suggest the author add one table of descriptive statistics (n, mean +/- standard error, confidence intervals for mean, min-max) for each category (types of organs, year of collection, age, sex).

Answer

Tables of descriptive statistics has been added (Table n. 2 and n. 3 from Page 31 Line 671 to Page 32 Line 693) or modified (Table n. 1 from Page 30 Line 663 to Line 669) as requested by reviewer 3.

(3) In the discussion, I would like the author to add the point that Cs-137 tends to show high accumulation in muscle due to its high potassium K content. Cesium (Cs) and Potassium (K) have some similarities in their biodynamic patterns because they belong to the same group in the periodic table, Group 1 (alkali metals), and due to the fact that muscle tissue, such as skeletal muscles, contain significant amounts of potassium for metabolic functions. Similar to Sr-90 and calcium (Ca). This seems to correlate with the findings of the work where muscle (diaphragm and tongue) show high activities. Please cite this using appropriate references as well as ICRP Publication 110 Realistic reference phantoms.

Answer

A new paragraph and two new references were added to highlight the suggested point (Page 17 from Line 385 to Line 391) and references n. 37 and 38 (from Page 27 Line 618 to Page 28 Line 625).

- AAVV (2010). Adult Reference Computational Phantoms, Annals of the International Commision on Radiological Protection, 39 (2), 170 pp;
- Large MJ, Malaroda A, Petasecca M, Rosenfeld AB; Guatelli S (2020) Modelling ICRP110 Adult Reference Voxex Phantoms for dosimetric application: Development of a new Geant4 Advanced Example. *J* *Pys:* *Conference* *Series* 1662, 012021 https://doi.org/10.1088/1742-6596/1662/1/012021;

Specific Comments:

Keywords: Keywords are crucial for increasing the visibility of the article and guiding readers. Consider including terms such as "Cesium 137-contaminated wild boar," "Cs-137 contaminated animal organ," "Cs-137 in Sus Scrofa," "wild boar radioactive bioindicator," and "North-western Italy Cesium-contamination."

Answer

The suggested keywords were included (Page 1 from Line 18 to Line 20).

Abstract: Limited values of results are given as most of the results are discussed. Add important values of the obtained results, e.g., mean, min-max based on different organ sites. This is important as the abstract needs to reflect the title, i.e., Cs in wild boar, but none of the Cs activity values are presented. Add a brief line about the main instrument used in this work. All approaches and methods are well described. Discussion and conclusion are sufficient.

Answer

Results (Page 2 from Line 39 to Line 41 and Page 3 from Line 44 to Line 48) and the instrument information (Page 2 from Line 34 to Line 37) were added to the abstract.

Introduction: Revise the introduction to reflect the revised title and aim of the study.

Answer

The introduction was revised to reflect the title and the aim of the study (from Page 6 Line 132 to Page 7 Line 138).

Study Site: Revise the map in Fig. 1. Please title the countries in the map.

Answer

The names of the countries were added to the map (Fig. n. 1).

Line 177 - please elaborate on the process of homogenization. What matrix of the sample was obtained in this work? Is it in wet weight?

Answer

The paragraph has been completed with the requested information (Page 9 from Line 185 to Line 191).

Line 229 - define ARPA, as it is missing in affiliation and not all readers recognize Agenzia Regionale per la Protezione dell'Ambiente.

Answer

ARPA was defined as requested (Agenzia Regionale per la Protezione dell'Ambiente) (Page 1 Line 10, Page 2 from Line 35 to line 36 and Page 13 from Line 291 to Line 292).

Line 252-258 - metric SI prefix for (10^3) k or K?

Answer

The requested changes have been done (Page 14 Line 315, Page 14 from Line 317 to Line 318 and Page 17 from Line 381 to Line 382).

Line 233 - superscript m^2

Answer

The requested changes have been done (Page 13 Line 296).

Line 245 - "Bq/k" does not appear to be a standard unit for specific activity, and it may be a typographical error.

Answer

The requested change has been done (Page 11 Line 247).

Line 296 - p significant symbol. Sometimes you use small "p" and sometimes you use capital "P." Please be consistent.

Answer

The requested changes have been done through the text (Page 11 Line 249 and Line 251, Page 12 Line 254 and Page 16 Line 350).

Line 319 - sometimes "137Cs," sometimes "137^Cs," be consistent.

Answer

The requested changes have been done through the text (Page 7 Line 138, Page 10 Line 223 and Line 226, Page 11 Line 233, Page 12 Line 265, Page 14 Line 308, Page 26 Line 580, Page 27 Lines 609, Page 29 line 647, Page 30 Line 665, Page 33 Line 701 and Fig. 2 caption).

Fig. 2 missing unit on the y-axis.

Answer

The unit has been added (Fig. 2).

Supplementary materials 1 & 2 - revise the title of each column. Typos and capital/small letter issues. The symbol for kilogram is "kg," not "Kg."

Answer

The requested changes have been done (S1 and S2).

That's all.


[Note: HTML markup is below. Please do not edit.]

Reviewers' comments:

Reviewer's Responses to Questions

**Comments to the Author**

1. If the authors have adequately addressed your comments raised in a previous round of review and you feel that this manuscript is now acceptable for publication, you may indicate that here to bypass the “Comments to the Author” section, enter your conflict of interest statement in the “Confidential to Editor” section, and submit your "Accept" recommendation.

Reviewer #3: All comments have been addressed

Reviewer #5: (No Response)

2. Is the manuscript technically sound, and do the data support the conclusions?

The manuscript must describe a technically sound piece of scientific research with data that supports the conclusions. Experiments must have been conducted rigorously, with appropriate controls, replication, and sample sizes. The conclusions must be drawn appropriately based on the data presented.

Reviewer #3: Yes

Reviewer #5: (No Response)

3. Has the statistical analysis been performed appropriately and rigorously?

Reviewer #3: I Don't Know

Reviewer #5: (No Response)

4. Have the authors made all data underlying the findings in their manuscript fully available?

The [PLOS Data policy](http://www.plosone.org/static/policies.action#sharing) requires authors to make all data underlying the findings described in their manuscript fully available without restriction, with rare exception (please refer to the Data Availability Statement in the manuscript PDF file). The data should be provided as part of the manuscript or its supporting information, or deposited to a public repository. For example, in addition to summary statistics, the data points behind means, medians and variance measures should be available. If there are restrictions on publicly sharing data—e.g. participant privacy or use of data from a third party—those must be specified.

Reviewer #3: Yes

Reviewer #5: (No Response)

5. Is the manuscript presented in an intelligible fashion and written in standard English?

PLOS ONE does not copyedit accepted manuscripts, so the language in submitted articles must be clear, correct, and unambiguous. Any typographical or grammatical errors should be corrected at revision, so please note any specific errors here.

Reviewer #3: Yes

Reviewer #5: (No Response)

6. Review Comments to the Author

Please use the space provided to explain your answers to the questions above. You may also include additional comments for the author, including concerns about dual publication, research ethics, or publication ethics. (Please upload your review as an attachment if it exceeds 20,000 characters)

Reviewer #3: (No Response)

Reviewer #5: (No Response)

7. PLOS authors have the option to publish the peer review history of their article ([what does this mean?](https://journals.plos.org/plosone/s/editorial-and-peer-review-process#loc-peer-review-history)). If published, this will include your full peer review and any attached files.


If you choose “no”, your identity will remain anonymous but your review may still be made public.


**Do you want your identity to be public for this peer review?** For information about this choice, including consent withdrawal, please see our [Privacy Policy](https://www.plos.org/privacy-policy).

Reviewer #3: No

Reviewer #5: **Yes:** Tibor Kovacs

[NOTE: If reviewer comments were submitted as an attachment file, they will be attached to this email and accessible via the submission site. Please log into your account, locate the manuscript record, and check for the action link "View Attachments". If this link does not appear, there are no attachment files.]

While revising your submission, please upload your figure files to the Preflight Analysis and Conversion Engine (PACE) digital diagnostic tool, <https://pacev2.apexcovantage.com/>. PACE helps ensure that figures meet PLOS requirements. To use PACE, you must first register as a user. Registration is free. Then, login and navigate to the UPLOAD tab, where you will find detailed instructions on how to use the tool. If you encounter any issues or have any questions when using PACE, please email PLOS at [figures@plos.org](mailto:figures@plos.org). Please note that Supporting Information files do not need this step.

*In compliance with data protection regulations, you may request that we remove your personal registration details at any time.* [*(Remove my information/details)*](https://www.editorialmanager.com/pone/login.asp?a=r)*. Please contact the publication office if you have any questions.*

|  | |  |
| --- | --- | --- |
|  |  | |
